# High prevalence of magnesium supplementation during pregnancy: results from the LIFE Child study

**DOI:** 10.1007/s00404-026-08522-z

**Published:** 2026-08-31

**Authors:** Martina P. Neininger, Sophie U. J. Bremer, Anne Dathan-Stumpf, Holger Stepan, Wieland Kiess, Astrid Bertsche, Mandy Vogel, Thilo Bertsche

**Affiliations:** 1 Drug Safety Centre, Clinical Pharmacy, Institute of Pharmacy, Medical Faculty, Leipzig University Hospital and Leipzig University, Leipzig University, Bruederstrasse 32, 04103 Leipzig, Germany; 2 Center for Children and Adolescents, Department of Neuropediatrics, University Medicine Greifswald, Greifswald, Germany; 3 Department of Obstetrics, University Hospital Leipzig, Leipzig, Germany; 4 LIFE Leipzig Research Centre for Civilization Diseases, Leipzig University, Leipzig, Germany; 5 University Hospital for Children and Adolescents, Centre for Pediatric Research, Leipzig, Germany

**Keywords:** Pregnancy, Supplementation, Magnesium, Pregnancy cohort, Premature contractions

## Abstract

**Purpose:**

We examined the prevalence of magnesium supplementation among pregnant women in Germany and assessed whether risk factors for preterm birth might be associated with magnesium use. We explored the association between magnesium supplementation and pregnancy duration .

**Methods:**

The LIFE Child Study (Leipzig, Germany) recruited pregnant women and collected data in the 24^th^ and 36^th^ week of pregnancy from 2011 to 2019. In this prospective observational cohort study (*n* = 1078 participants), we analysed potential associations for magnesium supplementation with health-related factors using logistic regression models adjusted for age and socioeconomic status (SES). Using a generalised linear model, we explored whether magnesium supplementation potentially affects pregnancy duration.

**Results:**

During their current pregnancy, 60.0% (647/1078) of the women supplemented mono-magnesium, and 70.8% (763/1078) supplemented magnesium through mono- and/or combination preparations (overall magnesium). In 215 women, information on physicians’ recommendations/prescriptions was collected: In 84.2% (181/215) of those women, a physician prescribed (104/215, 48.4%) or recommended (77/215, 35.8%) the mono-magnesium supplement. Increasing age was positively associated with magnesium supplementation with overall magnesium use increasing from 57.8% at age 20 years to 84.3% at age 45. Women with medium (OR 2.93; 95% CI 1.20–7.37) or high SES (OR 3.82; 95% CI 1.54–9.77) were more likely to supplement overall magnesium than women with low SES. Mono-magnesium supplementation was more likely in women with premature contractions (OR 2.97; 95% CI 1.36–7.47) with a still positive but not significant effect for overall magnesium (OR = 1.78, 95% CI 0.81–4.49). Magnesium supplementation did not affect pregnancy duration.

**Conclusion:**

Magnesium supplementation during pregnancy is widespread. Increasing age, higher SES, and premature contractions seem to have the potential to influence magnesium supplementation during pregnancy.

**Trial registration:**

LIFE Child is registered under the trial number: NCT02550236.

**Supplementary Information:**

The online version contains supplementary material available at https://doi.org/10.1007/s00404-026-08522-z.

## What does this study add to the clinical work?

**Table:** 

Magnesium supplementation is highly prevalent among pregnant women in Germany and is to a considerable extent based on a physician’s recommendation or prescription. Associations were observed for only a few health-related factors such as premature contractions or increasing age; for most pregnant women, no association with health-related factors was found.	

## Introduction

Vitamin and mineral supplementation is widespread in Western countries [1], particularly during pregnancy, when micronutrient requirements increase. In countries such as Germany, these increased needs can generally be met through a balanced diet. Consequently, supplementation is recommended for only a limited number of micronutrients, such as folic acid, but not magnesium [2–4]. However, commercial information and advertising frequently emphasise potential benefits of magnesium also for pregnant women and suggest increased requirements during pregnancy, although this is not supported by national dietary recommendations. The recommended daily intake of magnesium (300mg) according to the European Food Safety Authority (EFSA) and the German Nutrition Society is identical for pregnant and non-pregnant women [5, 6]. This amount can be ingested through a diet containing, e.g., whole grains, pulses, leafy green vegetables, nuts and seeds, potatoes, bananas, meat, and milk and dairy products [6]. However, it remains unclear whether healthcare professionals and pregnant women are aware that daily requirements of magnesium can be met through diet, given the absence of regular standardised nutrition education for pregnant women in Germany.

Oral magnesium supplementation has been hypothesised to reduce the risk of preterm birth, based largely on findings from a magnesium-deficient region [7]. However, current evidence does not support a general recommendation for oral magnesium supplementation, as clear benefits have not been demonstrated and further high-quality studies are required [8, 9]. This is particularly relevant in Germany, where magnesium deficiency is rare [10].

Data on magnesium supplementation during pregnancy are scarce. This study aimed to assess the frequency of magnesium supplementation during pregnancy and to examine socioeconomic determinants. In addition, we sought to explore whether women with risk factors for preterm birth might be more likely to supplement magnesium and whether supplementation might be associated with pregnancy duration. To address these objectives, we conducted an exploratory data analysis of the Leipzig Research Centre for Civilization Diseases (LIFE) Child pregnancy cohort, a population-based cohort of pregnant women from the Leipzig region (Saxony, Germany).

## Methods

### Setting and study population

Our study was based on the LIFE Child study pregnancy cohort, consisting of women recruited during the second trimester [11, 12]. Pregnant women without any chronic, chromosomal, and syndromal diseases living in the Leipzig area (Saxony, Germany) were invited to participate by posters and leaflets at hospitals, gynaecologists’ offices, midwives’ offices, nursery schools, and the student information centre at Leipzig University. To make participation more appealing, pregnant women were offered detailed examinations and monetary compensation [11, 12]. The assessments were conducted in the 24^th^ (23 + 1 to 24 + 0, with an allowed time delay up to 25 + 6) and 36^th^ week of gestation (35 + 1 to 36 + 0), as defined in the study protocol.

The pregnancies we evaluated in the present analyses were continuously registered from the start of the study in May 2011 until December 2019 to obtain a large sample size. All participants gave written informed consent. The LIFE Child study was approved by the local Ethics Committee.

To minimise confounding, twin pregnancies were excluded. Both first pregnancies and subsequent pregnancies were included.

### Assessment of magnesium supplementation from medication data

During the examinations in the 24^th^ and 36^th^ week throughout the study period from 2011 to 2019, current medication and dietary supplementation were assessed in an interview. The participants were asked to bring the packages or a list of all preparations they took during the last two weeks. If the packaging or list was forgotten, study nurses or physicians called the participants for a follow-up phone interview. In 2016, a structured interview was implemented into the study protocol at the examination in the 24^th^ week to gain deeper insights and to collect additional information about whether a physician prescribed or recommended magnesium, and the duration of supplementation. Additionally, a food frequency and a pregnancy questionnaire were used to collect data concerning magnesium supplementation and health-related factors. We did not analyse the food intake by the pregnant women. Clinical or biochemical indicators of magnesium deficiency were not assessed. The questionnaire did also not address the motivations and reasons for or against magnesium supplementation.

Mono-magnesium preparations were defined as preparations containing magnesium as the main component. Those preparations either contained only magnesium, were called ‘magnesium’ by the interviewee, or were identified as a preparation with only a small quantity of additional components (i.e., other vitamins or minerals in small amounts). Women also supplemented multi-micronutrient preparations that contained magnesium. Whether a multi-micronutrient preparation contained magnesium was gathered from the nutritional information of the product. The daily dosage was not obtained, however, the magnesium amount per dose unit was documented.

According to the data on magnesium supplementation in the 24^th^ and 36^th^ gestational week, we grouped the pregnant women into participants with or without magnesium supplementation. When data were only available on one of the two appointments, we used the supplementation data from this appointment.

To explore potential associations between magnesium supplementation and socioeconomic and health-related factors, we categorised the magnesium supplementation into mono and overall supplementation. In the analyses for mono-magnesium supplementation, we excluded multi-micronutrient supplements containing magnesium, because multi-micronutrient supplements are presumably aimed at general supplementation with a variety of vitamins and minerals. The same analyses were carried out for overall magnesium supplementation including all supplements containing magnesium (mono-magnesium and magnesium containing multi-micronutrient supplements).

### Assessment of diseases and risk factors for preterm birth

To investigate associations between magnesium supplementation and risk factors for preterm birth (pregnancy duration < 37 + 0 weeks of gestation), we evaluated data from the German maternity pass, interviews conducted at 24^th^ and/or 36^th^ gestational week, and retrospective pregnancy questionnaires. The maternity pass is a document every pregnant woman in Germany receives at pregnancy diagnosis, in which all relevant data on medical history and prenatal care is documented [13]. These data include information about potential risk factors for preterm birth according to Vogel et al. such as (gestational) diabetes (diagnosed using the International Association of the Diabetes and Pregnancy Study Groups [IADPSG] criteria for the oral glucose tolerance test), obesity (pre-pregnancy BMI ≥ 30 kg/m^2^), hypertension (systolic blood pressure ≥ 140 mmHg, diastolic blood pressure ≥ 90 mmHg), infections during pregnancy, assisted pregnancy induction, short inter-pregnancy interval (< 12 months), premature contractions, or preterm birth in a previous pregnancy [14]. These risk factors were assessed by the women’s attending physicians and documented in the maternity pass.

### Assessment of socioeconomic status, age, and other relevant characteristics

Socioeconomic status was assessed using the Winkler–Stolzenberg score, a composite score combining information on education and occupational status of the pregnant woman and partner and the equivalised disposable household income. The socioeconomic status was categorised into the following categories: low (3–8.4 points), medium (8.5–15.4 points), and high (15.5–21 points) [15, 16]. To standardise the age of pregnant women, we used age at delivery for the calculations. Whether the pregnancy was planned or not was evaluated through a question that was part of an interview. Number of pregnancies and births was assessed through the maternity pass and from interviews.

### Statistics

Data analyses were performed using SPSS (IBM, Version 28) and R (Version 4.6.0). To analyse for associated factors, we ran logistic regressions for the outcome of magnesium supplementation. We considered the following variables in our dataset as possible associated factors, as these have been identified as risk factors for preterm birth: premature contractions, gestational diabetes, hypertension, obesity, infections during pregnancy, short inter-pregnancy interval, preterm birth in an earlier pregnancy, and assisted pregnancy induction [14]. We adjusted the logistic regression for age and socioeconomic status. The results are presented as estimated percentages with 95% confidence interval (95% CI) and odds ratios (OR) with 95% CI. We conducted these analyses for the mono-magnesium supplements and overall magnesium supplementation. No global correction for multiple comparisons was applied, as the predictors are clinically related, address a common underlying hypothesis, and cannot be considered statistically independent. Results should be interpreted in the context of the overall pattern of findings rather than as isolated *p* values.

To investigate a potential association between pregnancy duration and magnesium supplementation, we used generalised additive models for location, shape, and scale regression models (GAMLSS) [17] with pregnancy duration (days) as outcome and magnesium supplementation and age (included as spline function) as predictors including also the interaction term. In addition, we adjusted for socioeconomic status (continuous variable). Because the outcome was a count variable measured in days with a left-skewed distribution, we initially based our approach on a Poisson distribution. However, testing revealed considerable underdispersion in the data. To correct for this, we utilised a double Poisson distribution, which dynamically unlinks the mean and the variance (unlike a standard Poisson distribution where these are assumed equal). GAMLSS was selected to implement this, as it allows for modelling all distribution parameters rather than just the centre of the distribution. One model was done for mono-magnesium and one for overall magnesium supplementation. Further details on the modelling are presented in Online Resource 1.

As data collection was based on information obtained in interviews, questionnaires, and documents the pregnant women brought with them, we were not able to obtain information for every single variable from every participant. For transparency, we report the number of pregnancies for which the respective information was obtained. Hence, the reported percentages refer to varying denominators. Only pregnancies without missing values in the variables under consideration were included in the respective regressions. Associations between magnesium supplementation and health-related factors are reported as odds ratios (OR) with 95% CI from logistic regression models adjusted for age and socioeconomic status. Since the prevalence of magnesium supplementation in this cohort substantially exceeds 10%, odds ratios will overestimate the magnitude of association relative to prevalence ratios and should not be interpreted as approximations of relative risk. Readers are, therefore, directed to the estimated percentages displayed in Figs. 2 and 3, which allow absolute differences between groups, resp. the age effect to be assessed directly and represent the more clinically interpretable summary of effect size. In general, we considered a *p* value ≤ 0.05 to indicate statistical significance. As the proportion of women with risk factors was very low in our cohort, we would like to stress the descriptive character of our results; therefore, causal relations cannot be inferred.

## Results

### Characteristics of the study population and magnesium supplementation

**Fig. 1:**
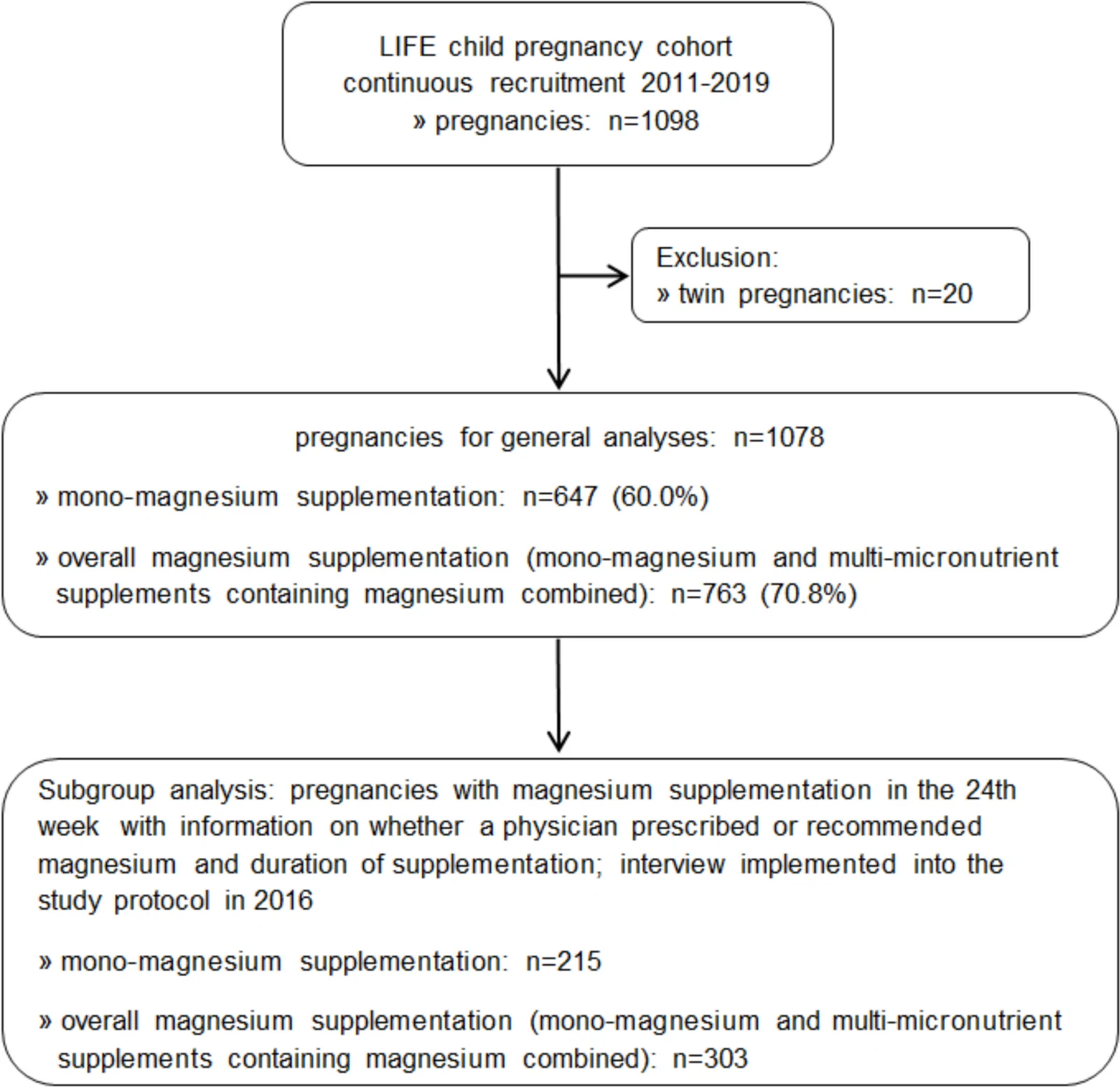
Participant flowchart

We included 1078 pregnancies in the analyses (Table 1, Fig. 1).

**Table 1:** Characteristics of the pregnant women

Characteristic	
Total number [*n*]	1078
Median gestational age at delivery (Q25; Q75) [weeks]	40 + 0 (39 + 0; 40 + 5) {*n* = 839}
Median age at birth (Q25; Q75) [years]	30.8 (28.3; 34.0) {*n* = 972}
Socioeconomic status (SES) by Winkler–Stolzenberg index	
Median SES (Q25; Q75)	15.0 (12.1; 16.8) {*n* = 1009}
SES low	2.4% (24/1009)
SES medium	58.4% (589/1009)
SES high	39.2% (396/1009)
Geographic origin of the pregnant woman: Germany	95.4% (860/901)
Median pre-pregnancy BMI (Q25; Q75) [kg/m^2^]	22.5 (20.7; 24.8) {*n* = 838}
Obesity (BMI ≥ 30 kg/m^2^)	7.6% (70/918)
Primigravida	45.7% (443/969)
Planned pregnancy	73.9% (690/934)
(Gestational) diabetes	6.5% (65/994)
Premature contractions	4.8% (48/991)
Hypertension	1.4% (14/1001)
Short inter-pregnancy interval (< 12 months)	7.3% (62/847)
Preterm birth in earlier pregnancy	3.1% (27/869)
Infections	4.4% (44/991)
Assisted pregnancy induction	4.7% (46/983)
Preterm birth (< 37.0 weeks of gestation)	4.5% (38/839)
Mono-magnesium supplementation	60.0% (647/1078)
Overall magnesium supplementation (mono-magnesium and multi-micronutrient supplements containing magnesium combined)	70.8% (763/1078)

*BMI* body mass index, (Gestational) Diabetes: diagnosed using the IADPSG criteria for the oral glucose tolerance test, Hypertension: systolic blood pressure ≥ 140 mmHg, diastolic blood pressure ≥ 90 mmHg; the number of missing values varies between the variables, and the numbers in curly brackets or in the denominator indicate the number of participants evaluated for the respective variable

Mono-magnesium supplements were taken by 60.0% (647/1078) and overall magnesium supplementation (mono-magnesium and multi-micronutrient supplements containing magnesium combined) by 70.8% (763/1078) of the women.

Regarding the initiation and the prescription/recommendation of magnesium supplementation, information in the 24^th^ week was obtained for the pregnant women who were recruited from 2016 to 2019. Of those women with mono-magnesium use, 62.8% (135/215) and of those with overall magnesium supplementation, 75.6% (229/303) began to take the supplement during pregnancy or no earlier than 6 months before pregnancy (Online Resource 2).

In 84.2% (181/215) of the women with mono-magnesium use, a physician prescribed (104/215, 48.4%) or recommended (77/215, 35.8%) the mono-magnesium supplement. The median amount of magnesium per dose unit was 100 mg (Q25; Q75 100 mg; 243 mg). 80.9% (174/215) of the women took the mono-magnesium supplement every day. The prevalence of mono-magnesium supplementation in the 24^th^ week did not differ between the periods 2016–2019 and 2011–2015 (*p* = 0.76). The characteristics of pregnancies with mono-magnesium supplementation with or without information on physician’s prescription or recommendation are shown in Online Resource 3.

### Logistic regression for factors related to magnesium supplementation

**Fig. 2:**
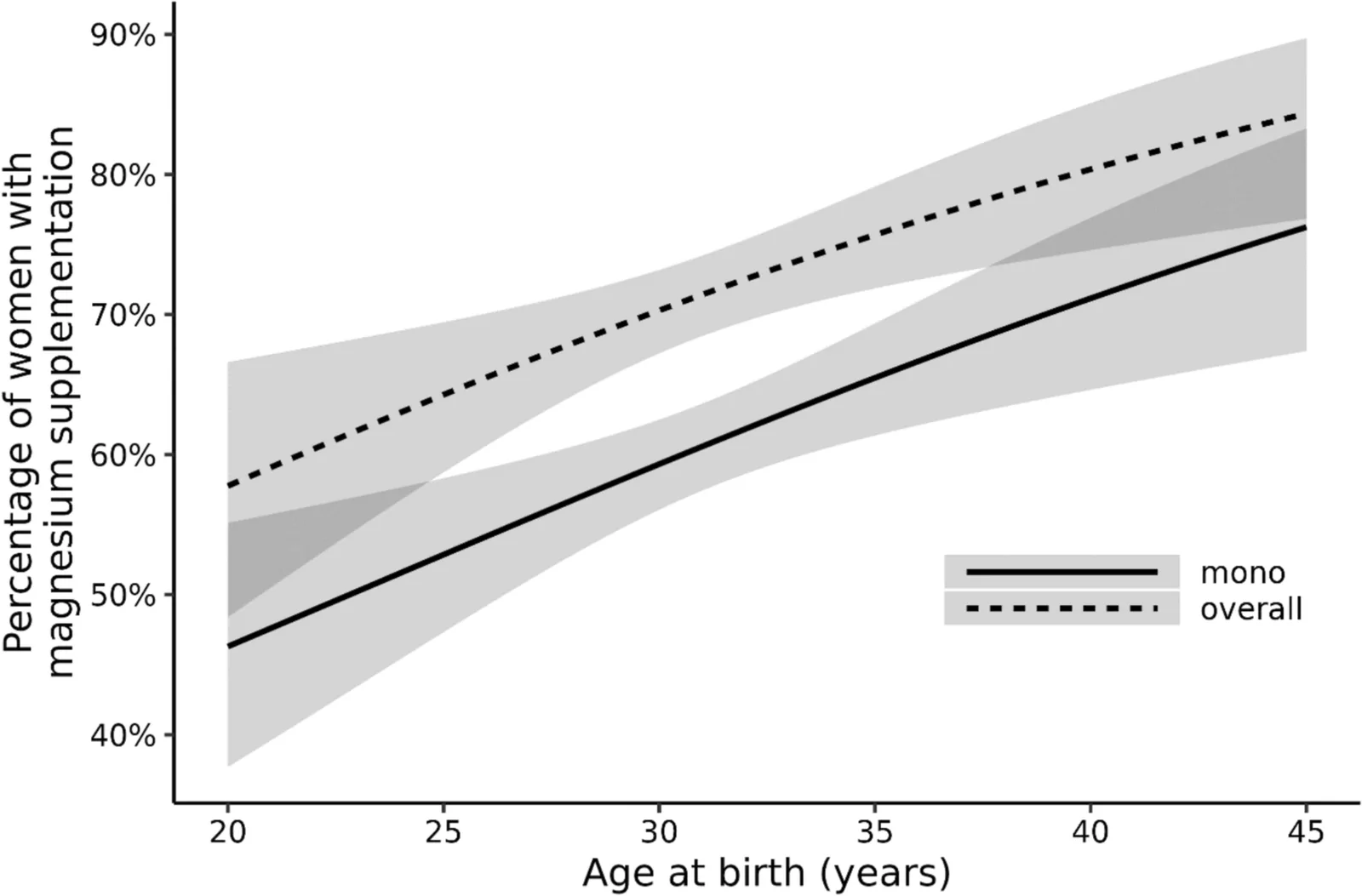
Positive association between age at birth and magnesium supplementation, estimated percentages and 95% CI, *mono* mono-magnesium supplementation, *overall* overall magnesium supplementation

The age of the pregnant women was positively associated with the intake of supplemental magnesium (*p* = 0.002). The lowest prevalence of magnesium supplementation was at the age of 20 years, 46.3% of the women were supplementing mono-magnesium and 57.8% overall magnesium. The highest prevalence was found at an age of 45 years, 76.2% of the women were supplementing mono-magnesium and 84.3% overall magnesium (Fig. 2).

**Fig. 3:**
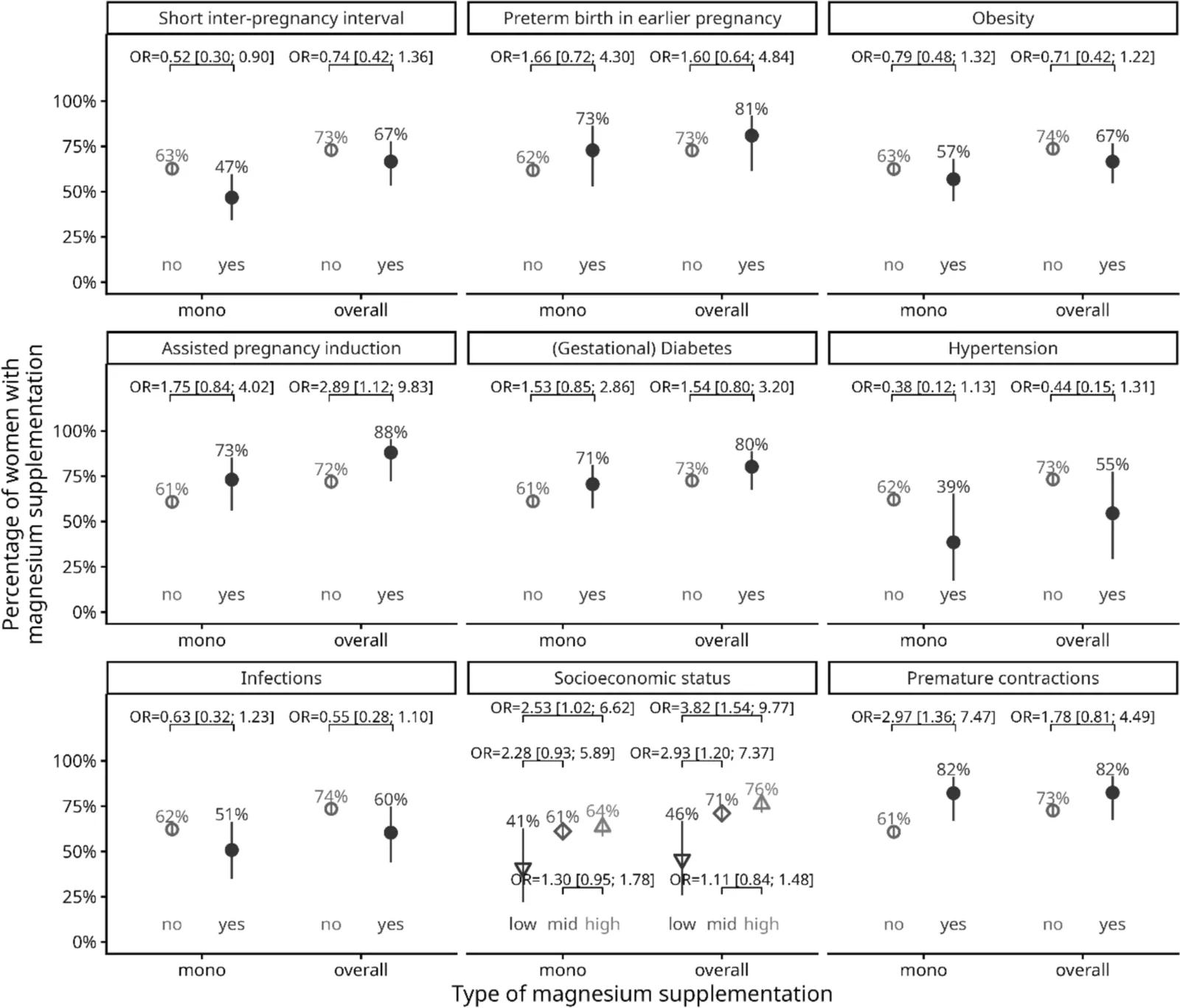
Potential associations of different factors for magnesium supplementation adjusted for age and socioeconomic status, estimated percentages and 95% CI, OR and 95% CI; analyses of socioeconomic status were only adjusted for age; *mono* mono-magnesium supplementation, *overall* overall magnesium supplementation

Given the associations between magnesium supplementation and age, we controlled for this variable in our exploratory analyses of possible relations between magnesium supplementation and risk factors for preterm birth. Women with a low socioeconomic status were less likely to supplement overall magnesium than women with a medium or high socioeconomic status, and were also less likely to supplement mono-magnesium than women with a high socioeconomic status. As socioeconomic status was identified as likely associated with magnesium supplementation, we also controlled for socioeconomic status in the further analyses. In women with premature contractions as diagnosed by their attending physician, mono-magnesium supplementation was more likely than in women without. We did not find this association for overall magnesium supplementation. In our exploratory analyses, we did not find an association of mono-magnesium or overall magnesium supplementation in women with hypertension, preterm birth in an earlier pregnancy, obesity, infections, or (gestational) diabetes (Fig. 3). Women with short inter-pregnancy interval were potentially less likely to supplement mono-magnesium and women with an assisted pregnancy induction were more likely to supplement overall magnesium.

Women without established risk factors for preterm birth (gestational diabetes, hypertension, obesity, premature contractions, history of preterm birth, obesity, infections, or assisted pregnancy induction) were not less likely to receive a physician’s prescription or recommendation for mono-magnesium supplementation than those with risk factors (*p* = 0.53, adjusted for age and socioeconomic status). Likewise, physician prescription or recommendation of mono-magnesium supplementation was not associated with socioeconomic status (*p* = 0.46, adjusted for age).

### Association of magnesium supplementation with pregnancy duration

No clinically meaningful or statistically significant association between magnesium supplementation and pregnancy duration was found at any maternal age (Online Resource 1). For overall magnesium supplementation, marginal effect estimates ranged from − 0.05 days at age 25 to − 2.11 days at age 40, with bootstrap 95% CIs spanning zero at all ages (age 25: − 1.99 to 2.09; age 30: − 1.98 to 0.61; age 35: − 2.88 to 0.03; age 40: − 4.27 to 0.14). For mono-magnesium supplementation, estimates were smaller and showed no age gradient (age 25: − 0.74 days, 95% bootstrap CI − 2.56 to 1.15; age 30: − 0.63 days, − 1.97 to 0.65; age 35: − 0.52 days, − 1.98 to 1.08; age 40: − 0.42 days, − 2.62 to 1.84).

## Discussion

Our analysis of pregnant women from a major city in Germany sheds light on magnesium supplementation and potentially associated factors. We found that supplemental magnesium is widely used during pregnancy. Mono-magnesium preparations were to a considerable extent prescribed or recommended by a physician. In women with risk factors for preterm birth mono-magnesium supplementation was not more likely with the exception of premature contractions compared to women without risk factors. Whether magnesium was taken or not did not result in a relevant difference in pregnancy duration in our analysis.

### Prevalence of magnesium supplementation

The prevalence of magnesium supplementation in our cohort is markedly higher than that reported in other populations, such as Polish studies showing supplementation rates of 5.6% and 12.4% [18, 19], or a cohort study from Quebec (Canada), in which magnesium was not among the most frequently used single-nutrient supplements [20]. These findings are particularly striking when compared with Poland, where magnesium supplementation is explicitly recommended for women with confirmed magnesium deficiency by the Polish Gynecological Society [21]. In contrast, general magnesium supplementation is not included in German nutritional recommendations for pregnant women [2], nor is it considered essential in dietary guidelines from other Western countries [3–5, 22]. Most women initiated magnesium supplementation during pregnancy or within the 6 months preceding conception. This suggests that magnesium supplementation was closely linked to pregnancy planning or pregnancy itself, despite the absence of guideline recommendations. However, the data of this study do not allow for an in-depth analysis of the pregnant women’s reasons or motivations to supplement magnesium.

We hypothesised that women with established risk factors for preterm birth would be more likely to receive a physician’s prescription or recommendation for a mono-magnesium supplement. In addition, magnesium is frequently prescribed during the first trimester to avoid an impending miscarriage if there are no other treatment options. However, our exploratory analyses did not reveal an association between risk factors for preterm birth and the prescription or recommendation of mono-magnesium supplements, despite the fact that 84% of mono-magnesium preparations were prescribed or recommended by a physician among women for whom information on physician’s prescription or recommendation was available.

These findings raise questions regarding the extent of physician-driven magnesium supplementation in the absence of a clear evidence-based indication, particularly given that current guidelines do not support routine magnesium supplementation during pregnancy. Considering that more than two-thirds of pregnant women in our study reported magnesium supplementation, this represents a substantial proportion of the pregnant population and highlights the need for increased attention to this issue by professional medical societies and healthcare providers. Given that shortcomings in healthcare professionals' counselling have also been demonstrated for other dietary supplements during pregnancy, counselling tools such as standardised checklists may be beneficial [23].

In addition, greater emphasis could be placed on promoting adequate magnesium intake through a balanced diet, both among healthcare professionals and pregnant women. Dietary sources of magnesium not only contribute to meeting magnesium requirements but also provide a wide range of other essential nutrients and bioactive plant compounds that may be beneficial during pregnancy.

### Magnesium supplementation, pregnancy duration, and risk factors for preterm birth

In our study, magnesium supplementation did not reveal a clinically meaningful main effect on pregnancy duration across the central range of maternal age. The directional age gradient visible in the overall magnesium model—supplementing women having marginally shorter pregnancies at older ages—is consistent with confounding by indication: older women carry higher baseline obstetric risk and are simultaneously more likely to have indicated early deliveries. This pattern is absent for mono-magnesium, suggesting that the age-dependent signal in the overall model is driven by multi-micronutrient supplement users, consistent with our finding that assisted pregnancy induction was associated with overall but not mono-magnesium supplementation. We interpret this as reflecting greater health-seeking behaviour and comprehensive supplement use among older higher-risk women rather than any pharmacological effect of magnesium on pregnancy duration.

Beneficial effects on pregnancy duration have primarily been reported in an older study conducted in a region with a high prevalence of magnesium deficiency [7]. Magnesium deficiency mainly occurs in individuals with insufficient intake or increased renal excretion, for example due to saluretic medication [24]. However, mild reductions in intake do not necessarily result in deficiency, and magnesium deficiency is uncommon in Germany [25]. Yet, data on magnesium deficiency particularly in pregnancy are hardly available. The women in our cohort were generally healthy, and the use of saluretic medication during pregnancy is rare [26]. Therefore, findings from magnesium-deficient populations are unlikely to be transferable to the German context. Our results are consistent with a Cochrane review that found no significant effects of magnesium supplementation on pregnancy duration or preterm birth rates [9].

Given that magnesium deficiency has been associated with a higher risk for hypertensive disorders, diabetes, and pre-eclampsia [27, 28]—known risk factors for preterm birth—one might expect higher supplementation rates among women with these conditions. In our study, however, only premature contractions were potentially associated with mono-magnesium use, possibly reflecting the widespread promotion of magnesium for muscle cramps and the prevention of premature contractions. Other risk factors, such as hypertension and gestational diabetes, were not associated with magnesium use. We observed a positive association between overall magnesium supplementation and assisted pregnancy induction, which may reflect increased health awareness and preventive use of multi-micronutrient supplements among these women.

### Socioeconomic status and magnesium supplementation

In Germany, mono-magnesium supplements are frequently reimbursed by health insurance when prescribed during pregnancy and are also inexpensive over the counter, which may explain the weak associations with socioeconomic status: there is no association when medium socioeconomic status is compared to low or high. Only when the extremes are compared, an association can be found. In contrast, overall magnesium supplementation that includes the use of more costly multi-micronutrient supplements containing magnesium that are not reimbursable, was considerably less likely in women with low socioeconomic status. This finding may reflect financial barriers to accessing supplements or differences in health-related behaviour across socioeconomic groups. It therefore raises important questions regarding health equity in the use of supplements during pregnancy. A study from the United States demonstrated a clear association between socioeconomic status and pregnancy outcomes [29]. In Germany, mono-ingredient supplements, such as folic acid, iron, and magnesium, are reimbursed by statutory health insurance when prescribed by a physician during pregnancy. However, this requires regular antenatal care attendance and prescribing practices independent of socioeconomic status. As no association between socioeconomic status and physician’s prescription or recommendation of mono-magnesium supplements was observed in our study, socioeconomic disparities might not play a major role specifically in mono-magnesium supplementation in Germany. Nevertheless, our data do not allow a comprehensive assessment of this issue as data on physician’s recommendation was not available in women who did not supplement magnesium at all. Further research is required, particularly regarding non-users and the use of multi-component supplements that are generally not reimbursed in Germany and are more likely to be used by women with medium or high socioeconomic status. In the case of magnesium, adequate intake may be achieved through dietary sources such as whole grains and or leafy green vegetables, which are broadly accessible and affordable also for individuals with low socioeconomic status. Even if a daily intake of up to 350 mg magnesium during pregnancy is assumed, as suggested by Italian and US guidelines [30], this amount can be achieved through dietary sources according to the German Nutrition Society [6].

## Limitations

First of all, our results are mainly of descriptive character and are not appropriate to infer causal associations rather than to explore potential relations that should be subject to further studies.

When data on magnesium supplementation were only available on one of the two appointments, we used the data from this appointment. Therefore, misclassification of women who supplemented magnesium at another time during pregnancy cannot be ruled out. Consequently, the prevalence of magnesium supplementation during pregnancy might even be higher in the analysed women. In addition, some participants were unable to specify exactly which product they had taken. This carries the risk that a product might have been incorrectly classified as ‘mono-magnesium’, even though it contains other ingredients. As potentially less supplement-aware women were more likely to be misclassified due to the higher probability of incomplete data on their supplement intake, a potential bias exists with regard to the analysis of the associations of magnesium supplementation and risk factors. This is further aggravated by the small number of women affected by risk factors in our cohort, limiting the statistical power to detect clinically meaningful associations. In addition, we only included cases without missing values in age, socioeconomic status, and information about the respective risk factor in the analysis of an association between magnesium supplementation and a given risk factor for preterm birth. Furthermore, several clinical predictors in our analysis—including documented premature contractions, infections, and gestational diabetes—are ascertainment-dependent. They are detected only through clinical encounters, and women with more frequent healthcare contact are more likely to have these conditions documented. This healthcare-contact confounding is compounded by the known selection bias in cohort studies towards higher socioeconomic status and greater health engagement. Therefore, the results of the analysis of the association between magnesium supplementation and risk factors have to be considered with caution, as they may also contain statistical artefacts or null findings despite actually existing associations.

Given the exploratory nature of these analyses and the number of statistical comparisons performed, the possibility of false-positive findings cannot be excluded. The association between premature contractions and mono-magnesium supplementation is the most consistent and substantively interpretable finding; the remaining associations, including the inverse association between short inter-pregnancy interval and mono-magnesium supplementation, should be interpreted with particular caution pending replication in independent cohorts.

As in other cohort-based studies, recruitment on a voluntary basis resulted in a sample with a higher socioeconomic status and healthier individuals than the general local population [31–33]. A higher socioeconomic status correlates with a higher health awareness including the intake of supplements. Additionally, almost all women were of German origin. As migrant women sometimes have poorer access to the healthcare system, our results could overestimate the prevalence of magnesium supplementation in the general German population. Altogether, this results in a selection bias towards a more health-aware cohort that is likely to be more homogeneous than the general German population. Thus, we assume that missing values are randomly distributed across the cohort.

Data on medical history and magnesium supplementation were obtained at the same assessment date. Hence, causal relationships between magnesium supplementation and the analysed symptoms and diseases cannot be drawn from our results. In addition, the questionnaire on physician’s prescription/recommendation was implemented into the study protocol in 2016, so data were available only for a subcohort. Although the health-related parameters are consistent between the cohorts with and without this data collection, the generalisability of this result is limited.

## Conclusions

In our study, almost two-thirds of pregnant women supplemented magnesium, a prevalence that is high compared with other Western countries. This is notable given that magnesium deficiency is uncommon in Germany and evidence supporting beneficial effects of oral supplementation remains inconclusive. Physicians nonetheless seem to play a central role with regard to mono-magnesium supplementation. Although a causal relationship cannot be inferred, we found a potential association between mono-magnesium supplementation and premature contractions.

## Supplementary Information

Below is the link to the electronic supplementary material.Supplementary file1 (PDF 835 KB)Supplementary file2 (PDF 51 KB)Supplementary file3 (PDF 96 KB)

## Data Availability

The datasets presented in this article are not readily available, because the data cannot be shared publicly due to ethical restrictions. The LIFE Child study is a study collecting potentially sensitive information. Publishing the datasets is not covered by the informed consent provided by the study participants. Furthermore, the LIFE data protection concept stipulates that all (external as well as internal) researchers interested in accessing the data sign a project agreement. Researchers interested in accessing and analysing data collected in the LIFE Child study may contact the data use and access committee (dm@life.uni-leipzig.de). Requests to access the datasets should be directed to dm@life.uni-leipzig.de.

## References

[1] Skeie G, Braaten T, Hjartåker A, Lentjes M, Amiano P, Jakszyn P, Pala V, Palanca A, Niekerk EM, Verhagen H, Avloniti K, Psaltopoulou T, Niravong M, Touvier M, Nimptsch K, Haubrock J, Walker L, Spencer EA, Roswall N, Olsen A, Wallström P, Nilsson S, Casagrande C, Deharveng G, Hellström V, Boutron-Ruault MC, Tjønneland A, Joensen AM, Clavel-Chapelon F, Trichopoulou A, Martinez C, Rodríguez L, Frasca G, Sacerdote C, Peeters PH, Linseisen J, Schienkiewitz A, Welch AA, Manjer J, Ferrari P, Riboli E, Bingham S, Engeset D, Lund E, Slimani N (2009) Use of dietary supplements in the European Prospective Investigation into Cancer and Nutrition calibration study. Eur J Clin Nutr 63(Suppl 4):S226-238. 10.1038/ejcn.2009.83 19888276

[2] Koletzko B, Cremer M, Flothkötter M, Graf C, Hauner H, Hellmers C, Kersting M, Krawinkel M, Przyrembel H, Röbl-Mathieu M, Schiffner U, Vetter K, Weißenborn A, Wöckel A (2018) Diet and lifestyle before and during pregnancy - practical recommendations of the Germany-wide Healthy Start - Young Family Network. Geburtshilfe Frauenheilkd 78:1262–1282. 10.1055/a-0713-1058 30655650 PMC6294644

[3] Procter SB, Campbell CG (2014) Position of the Academy of Nutrition and Dietetics: nutrition and lifestyle for a healthy pregnancy outcome. J Acad Nutr Diet 114:1099–1103. 10.1016/j.jand.2014.05.005 24956993

[4] Health Canada (2019) Canada’s dietary guidelines for health professionals and policy makers. Health Canada = Santé Canada, Ottawa, ON

[5] EFSA Panel on Dietetic Products Nutrition and Allergies (NDA) (2009) Scientific Opinion on the Substantiation of Health Claims Related to Magnesium and Electrolyte Balance (ID 238), Energy-yielding Metabolism (ID 240, 247, 248), Neurotransmission and Muscle Contraction Including Heart Muscle (ID 241, 242), Cell Division (ID 365), Maintenance of Bone (ID 239), Maintenance of Teeth (ID 239), Blood Coagulation (ID 357) and Protein Synthesis (ID 364) Pursuant to Article 13 (1) of Regulation (EC) no 1924/2006. EFSA J 7(9):1216

[6] German Nutrition Society. Magnesium. https://www.dge.de/wissenschaft/referenzwerte/magnesium/. Accessed 29 May 2026

[7] Wynn A, Wynn M (1988) Magnesium and other nutrient deficiencies as possible causes of hypertension and low birthweight. Nutr Health 6:69–88. 10.1177/026010608800600201 3072500

[8] Han S, Crowther CA, Moore V (2013) Magnesium maintenance therapy for preventing preterm birth after threatened preterm labour. Cochrane Database Syst Rev 2103:CD000940. 10.1002/14651858.CD000940.pub3 PMC7063385 23728634

[9] Makrides M, Crosby DD, Bain E, Crowther CA (2014) Magnesium supplementation in pregnancy. Cochrane Database Syst Rev 2014(4):CD000937. 10.1002/14651858.CD000937.pub2 24696187 PMC6507506

[10] Mensink GBM, Fletcher R, Gurinovic M, Huybrechts I, Lafay L, Serra-Majem L, Szponar L, Tetens I, Verkaik-Kloosterman J, Baka A, Stephen AM (2013) Mapping low intake of micronutrients across Europe. Br J Nutr 110:755–773. 10.1017/S000711451200565X 23312136 PMC3785176

[11] Quante M, Hesse M, Döhnert M, Fuchs M, Hirsch C, Sergeyev E, Casprzig N, Geserick M, Naumann S, Koch C, Sabin MA, Hiemisch A, Körner A, Kiess W, LIFE Child Study Investigators (2012) The LIFE child study: a life course approach to disease and health. BMC Public Health 12:1021. 10.1186/1471-2458-12-1021 23181778 PMC3533937

[12] Poulain T, Baber R, Vogel M, Pietzner D, Kirsten T, Jurkutat A, Hiemisch A, Hilbert A, Kratzsch J, Thiery J, Fuchs M, Hirsch C, Rauscher FG, Loeffler M, Körner A, Nüchter M, Kiess W, Child LIFE, study team, (2017) The LIFE Child study: a population-based perinatal and pediatric cohort in Germany. Eur J Epidemiol 32:145–158. 10.1007/s10654-016-0216-9 28144813

[13] Gemeinsamer Bundesausschuss. Richtlinien des Gemeinsamen Bundesausschusses über die ärztliche Betreuung während der Schwangerschaft und nach der Geburt. In: Bundesanzeiger AT 12.05.2023 B4; last change 16 February 2023; entered into force on 13 May 2023

[14] Vogel JP, Chawanpaiboon S, Moller AB, Watananirun K, Bonet M, Lumbiganon P (2018) The global epidemiology of preterm birth. Best Pract Res Clin Obstet Gynaecol 52:3–12. 10.1016/j.bpobgyn.2018.04.003 29779863

[15] Lampert T, Müters S, Stolzenberg H, Kroll LE (2014) Messung des sozioökonomischen Status in der KiGGS-Studie: Erste Folgebefragung (KiGGS Welle 1). [Measurement of socioeconomic status in the KiGGS study: first follow-up (KiGGS Wave 1)]. Bundesgesundheitsblatt Gesundheitsforschung Gesundheitsschutz 57:762–770. 10.1007/s00103-014-1974-8 24950825

[16] Lampert T, Hoebel J, Kuntz B, Müters S, Kroll LE (2018) Socioeconomic status and subjective social status measurement in KiGGS Wave 2. J Health Monit 3:108–125. 10.17886/RKI-GBE-2018-033 35586179 PMC8848848

[17] Rigby RA, Stasinopoulos MD, Heller GZ, de Bastiani F (2020) Distributions for modelling location, scale, and shape: using GAMLSS in R, 1st edn. CRC Press, Bocat Raton, London, New York

[18] Jankowska A, Grzesiak M, Krekora M, Dominowska J, Jerzyńska J, Kałużny P, Wesołowska E, Szadkowska-Stańczyk I, Trafalska E, Kaleta D, Kowalska M, Jabłońska E, Janasik B, Gromadzińska J, Hanke W, Wąsowicz W, Calamandrei G, Polańska K (2021) Determinants of the essential elements and vitamins intake and status during pregnancy: a descriptive study in Polish mother and child cohort. Nutrients 13:949. 10.3390/nu13030949 33809457 PMC8001522

[19] Kocyłowski R, Lewicka I, Grzesiak M, Gaj Z, Sobańska A, Poznaniak J, von Kaisenberg C, Suliburska J (2018) Assessment of dietary intake and mineral status in pregnant women. Arch Gynecol Obstet 297:1433–1440. 10.1007/s00404-018-4744-2 29541858 PMC5945726

[20] Dubois L, Diasparra M, Bédard B, Colapinto CK, Fontaine-Bisson B, Morisset AS, Tremblay RE, Fraser WD (2017) Adequacy of nutritional intake from food and supplements in a cohort of pregnant women in Québec, Canada: the 3D cohort study (Design, Develop, Discover). Am J Clin Nutr 106:541–548. 10.3945/ajcn.117.155499 28615265

[21] Karowicz-Bilinska A, Nowak-Markwitz E, Opala T, Oszukowski P, Poreba R, Spaczynski M (2014) Rekomendacje Polskiego Towarzystwa Ginekologicznego w zakresie stosowania witamin i mikroelementów u kobiet planujących ciążę, ciężarnych i karmiących. Ginekol Pol 85:395–399 25011224

[22] Australian Government Department of Health and Aged Care (2021) Nutrition advice during pregnancy. https://www.health.gov.au/resources/publications/nutrition-advice-during-pregnancy?language=en. Accessed 5 Feb 2026

[23] Hentrich AE, Brueggmann D, Deuster E, Kämpf AK, Jennewein L, Schaarschmidt W, Louwen F, Hoock SC (2025) Knowledge, awareness and recommendation on micronutrition during pregnancy-a survey of healthcare providers. Arch Gynecol Obstet 312:1155–1161. 10.1007/s00404-025-08111-6 40637878 PMC12414066

[24] Ryan MP, Devane J, Ryan MF, Counihan TB (1984) Effects of diuretics on the renal handling of magnesium. Drugs 28:167–181. 10.2165/00003495-198400281-00017 6389077

[25] Max Rubner Institute (2008) Nationale Verzehrsstudie II - Ergebnisbericht Teil 2. https://www.bmleh.de/SharedDocs/Downloads/DE/_Ernaehrung/NVS_ErgebnisberichtTeil2.html. Accessed 5 Feb 2026

[26] Schaefer C, Peters PWJ, Miller RK (eds) (2015) Drugs during pregnancy and lactation: treatment options and risk assessment. Elsevier/Academic Press, London

[27] Schoenaker DAJM, Soedamah-Muthu SS, Mishra GD (2014) The association between dietary factors and gestational hypertension and pre-eclampsia: a systematic review and meta-analysis of observational studies. BMC Med 12:157. 10.1186/s12916-014-0157-7 25241701 PMC4192458

[28] Wu J, Xun P, Tang Q, Cai W, He K (2017) Circulating magnesium levels and incidence of coronary heart diseases, hypertension, and type 2 diabetes mellitus: a meta-analysis of prospective cohort studies. Nutr J 16:60. 10.1186/s12937-017-0280-3 28927411 PMC5606028

[29] Nicholls-Dempsey L, Badeghiesh A, Baghlaf H, Dahan MH (2023) How does high socioeconomic status affect maternal and neonatal pregnancy outcomes? A population-based study among American women. Eur J Obstet Gynecol Reprod Biol X 20:100248. 10.1016/j.eurox.2023.100248 37876770 PMC10590715

[30] Mazza E, Maurotti S, Ferro Y, Castagna A, Pujia C, Sciacqua A, Pujia A, Montalcini T (2025) Magnesium: exploring gender differences in its health impact and dietary intake. Nutrients 17:2226. 10.3390/nu17132226 40647330 PMC12251677

[31] Heinrich J, Brüske I, Schnappinger M, Standl M, Flexeder C, Thiering E, Tischer C, Tiesler CM, Kohlböck G, Wenig CM, Bauer CP, Schaaf B, von Berg A, Berdel D, Krämer U, Cramer C, Lehmann I, Herbarth O, Behrendt H, Ring J, Kühnisch J, Koletzko S (2012) Die zwei deutschen Geburtskohorten GINIplus und LISAplus (Two German Birth Cohorts: GINIplus and LISAplus). Bundesgesundheitsblatt Gesundheitsforschung Gesundheitsschutz 55:864–874. 10.1007/s00103-012-1485-4 22736169

[32] Jacobsen TN, Nohr EA, Frydenberg M (2010) Selection by socioeconomic factors into the Danish National Birth Cohort. Eur J Epidemiol 25:349–355. 10.1007/s10654-010-9448-2 20349116

[33] Jaddoe VWV, van Duijn CM, van der Heijden AJ, Mackenbach JP, Moll HA, Steegers EA, Tiemeier H, Uitterlinden AG, Verhulst FC, Hofman A (2010) The generation R study: design and cohort update 2010. Eur J Epidemiol 25:823–841. 10.1007/s10654-010-9516-7 20967563 PMC2991548

