# Supplementary material for: High prevalence of magnesium supplementation during pregnancy: results from the LIFE Child study
Source: Arch Gynecol Obstet. 2026 Aug 31;313(1):261. doi: 10.1007/s00404-026-08522-z (PMC13529859; doi:10.1007/s00404-026-08522-z)
Supplement: Supplementary file 1 [file 404_2026_8522_MOESM1_ESM.pdf]

High prevalence of magnesium supplementation during pregnancy: results from the LIFE Child study

Archives of Gynecology and Obstetrics

Neininger*, Martina P[1,2]; Bremer*, Sophie UJ[1]; Dathan-Stumpf, Anne[3]; Stepan, Holger[3]; Kiess, Wieland[4,5]; Bertsche, Astrid[2,5]; Vogel, Mandy[4#]; Bertsche, Thilo[1#]

*) shared first authorship; #) shared senior authorship

Corresponding author:

Thilo Bertsche, Clinical Pharmacy, Institute of Pharmacy, Faculty of Medicine, Leipzig University and Drug Safety Centre, University Hospital Leipzig and Leipzig University, Bruederstrasse 32, 04103 Leipzig, Germany; email to thilo.bertsche@uni-leipizig.de, phone +49 341 97 11800; Fax number: +49 341 97 11849

Online Resource 1a Analysis of association between magnesium supplementation and pregnancy duration: detail on statistical methods

Pregnancy duration (days) was modelled using generalised additive models for location, scale, and shape (GAMLSS) with a Box-Cox Power Exponential (BCPE) distribution, selected on the basis of worm plot diagnostics which indicated that the initially fitted double Poisson distribution did not adequately capture the kurtosis of the residual distribution. Separate models were estimated for overall magnesium supplementation and mono-magnesium supplementation. In both models, the mean (mu) was specified as a function of magnesium supplementation, maternal age (penalized spline, pb()), socioeconomic status (continuous Winkler-Stolzenberg score), and an interaction between magnesium supplementation and maternal age. For the overall magnesium model, the dispersion parameter (sigma) was modelled as a function of magnesium supplementation and maternal age, and the shape parameter (nu) as a function of magnesium supplementation. For the mono-magnesium model, both sigma and nu were modelled as functions of mono-magnesium supplementation only; including maternal age in the sigma submodel produced non-estimable analytic standard errors, confirmed as a structural limitation of the BCPE variance-covariance approximation under penalized spline interactions rather than a numerical artefact. Models were fitted with a maximum of 50 cycles to ensure convergence. Marginal effects of magnesium supplementation on pregnancy duration were estimated at maternal ages 25, 30, 35, and 40 years. Since analytic standard errors were unreliable, inference was based exclusively on non-parametric bootstrap validation (500 resamples with replacement). The BCPE distribution models location (mu), scale (sigma), shape (nu), and kurtosis (tau) as distributional parameters; modelling nu as a function of magnesium supplementation allows the shape of the pregnancy duration distribution to differ between supplementing and non-supplementing women. Model adequacy was confirmed via different diagnostic plots including worm plots, which showed no systematic deviation from the assumed distributional family in either model.

Online Resource 1b Analysis of association between magnesium supplementation and pregnancy duration: generalised additive models for location, scale, and shape (GAMLSS) with a Box-Cox Power Exponential (BCPE) distribution marginal effects and validation

Marginal effects of magnesium supplementation on pregnancy duration (days) were estimated at four maternal ages. A positive estimate indicates longer predicted pregnancy duration in supplementing compared with non-supplementing women; a negative estimate indicates shorter predicted duration. Model-based standard errors for models containing penalized spline interactions with the BCPE family are known to be potentially unreliable due to limitations of the analytic variance-covariance approximation. Therefore, we present in addition bootstrap-based confidence intervals from non-parametric resampling (500 iterations, sampling with replacement from the complete-case analytic dataset).

Overall magnesium supplementation:

	Maternal age	estimate	conf.low	conf.high	p.value
1	25.00	0.09	-1.99	2.16	0.93
2	30.00	-0.56	-1.97	0.86	0.44
3	35.00	-1.20	-2.81	0.42	0.15
4	40.00	-1.84	-4.31	0.63	0.14

Bootstrap

	Maternal age	mean_est	ci_low	ci_high
1	25	-0.05	-1.99	2.09
2	30	-0.73	-1.98	0.61
3	35	-1.42	-2.88	0.03
4	40	-2.11	-4.27	0.14

Mono-magnesium supplementation:

	Maternal age	estimate	conf.low	conf.high	p.value
1	25.00	-0.18	-2.13	1.77	0.86
2	30.00	-0.15	-1.49	1.20	0.83
3	35.00	-0.11	-1.67	1.44	0.89
4	40.00	-0.08	-2.46	2.29	0.95

Bootstrap

	Maternal age	mean_est	ci_low	ci_high
1	25	-0.73	-2.56	1.15
2	30	-0.63	-1.97	0.65
3	35	-0.52	-1.98	1.08
4	40	-0.42	-2.62	1.84

Online Resource 1c Analysis of association between overall magnesium supplementation and pregnancy duration: model quality - worm plots

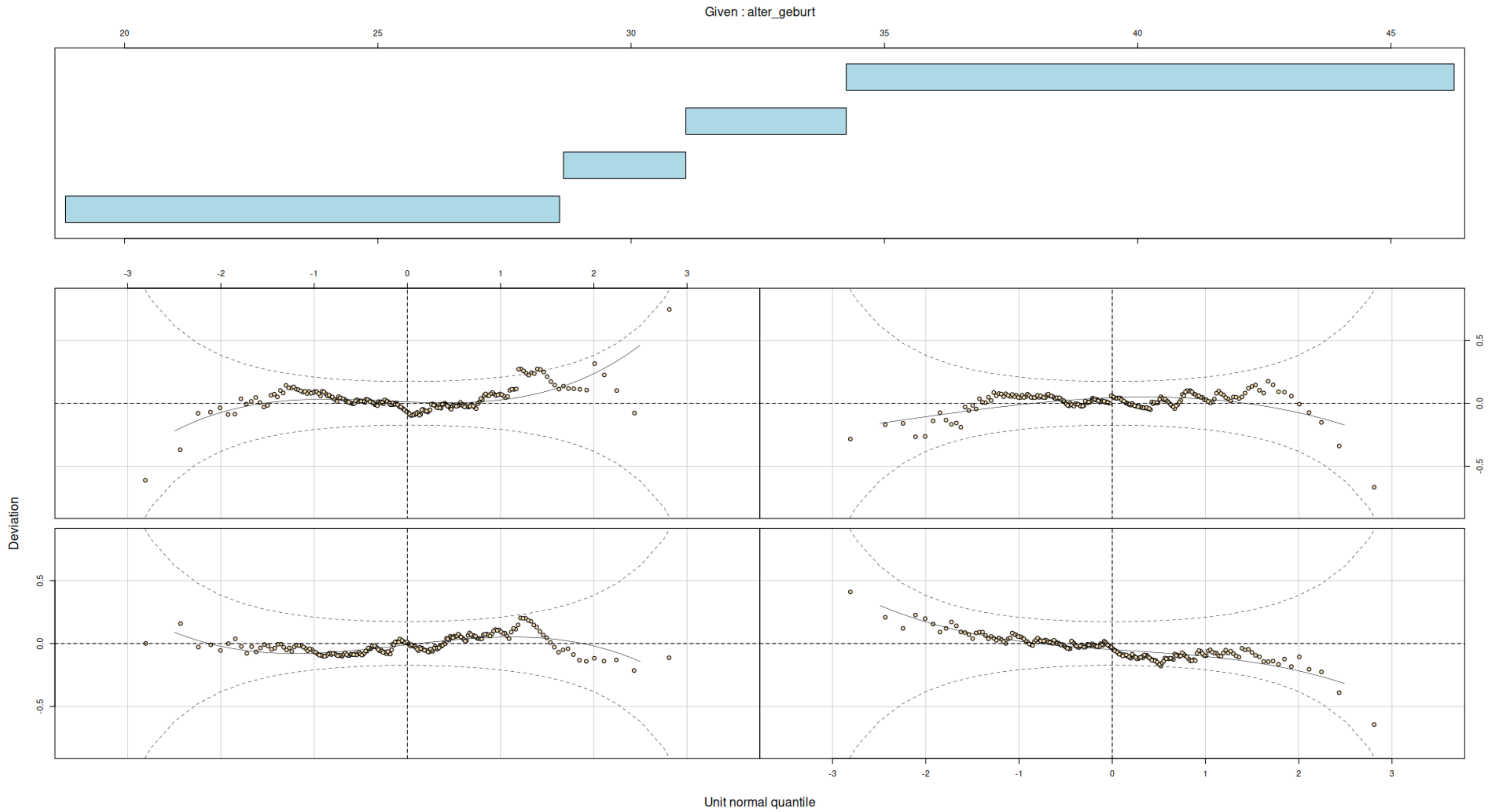

Worm plots (detrended Q-Q plots of randomised quantile residuals) from the GAMLSS model for overall magnesium supplementation, stratified by maternal age at delivery (binned into four intervals: approximately 20–25, 25–30, 30–35, and 35–45 years; bin widths are proportional to the number of observations, as shown in the bar chart above). The bar chart indicates the relative sample size within each age stratum, illustrating the substantially smaller number of observations in the oldest age bin. Worms fluctuating around zero within the reference ellipses indicate adequate fit; systematic S-shaped or U-shaped deviations would indicate skewness or kurtosis misspecification respectively. All four bins show worms remaining close to zero without systematic directional deviation, supporting the adequacy of the BCPE distributional assumption across the full age range. A modest upward drift is visible in the positive quantile range of the younger age strata (approximately 20–30 years), suggesting a slight tendency toward positive skewness in the residuals at lower fitted values. This deviation remains within or close to the reference ellipses and does not indicate substantial misspecification; it most plausibly reflects the left truncation of pregnancy duration in this age group, where very short gestations are rare and the distribution is consequently compressed at the lower tail. [alter_geburt: maternal age at delivery]

Online Resource 1d Analysis of association between mono-magnesium supplementation and pregnancy duration: model quality - worm plots

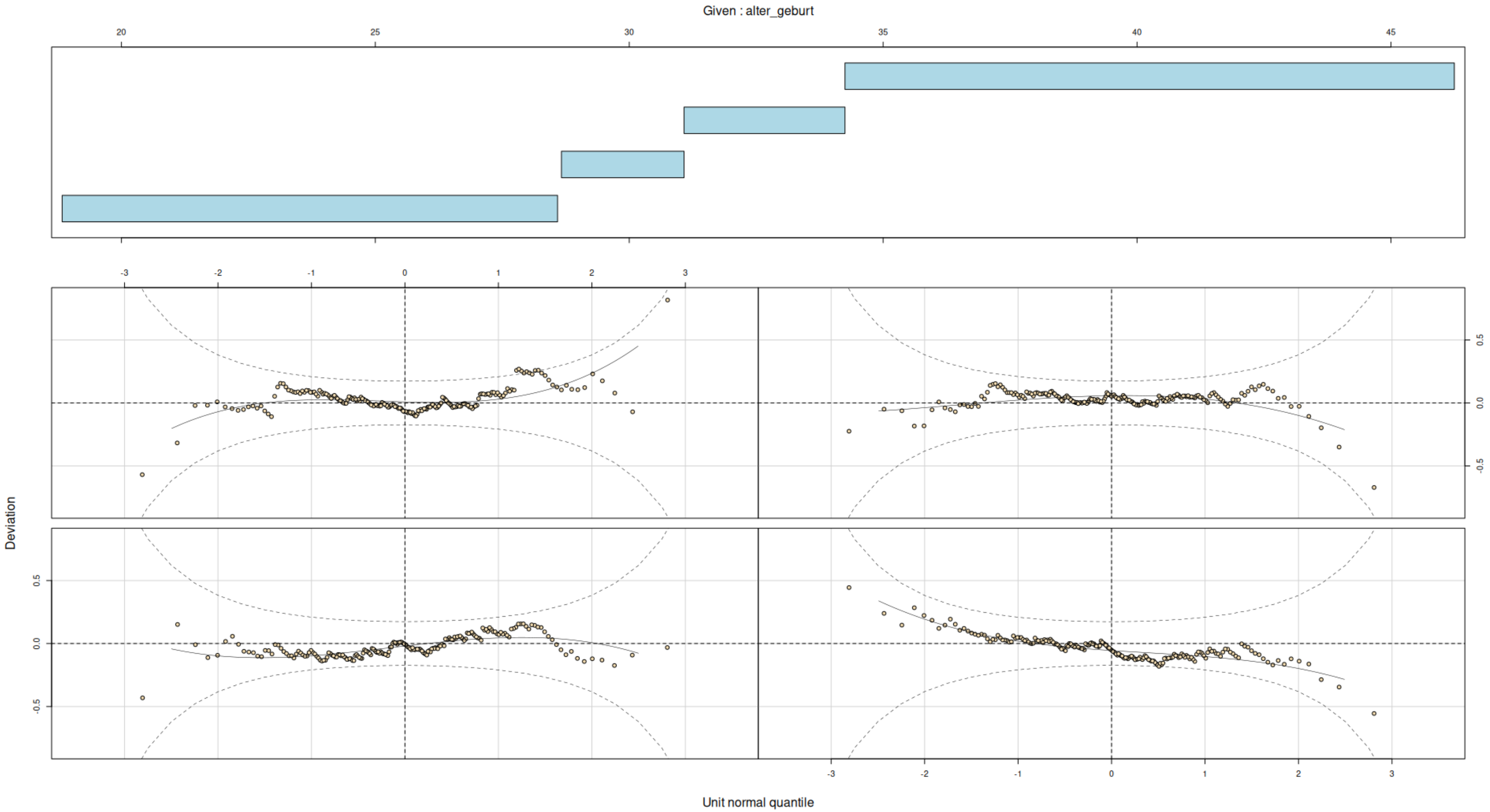

Worm plots from the GAMLSS model for mono-magnesium supplementation, stratified by maternal age at delivery. The overall pattern closely mirrors that of the overall magnesium model. Worms remain within or close to the reference ellipses across all age strata without systematic S-shaped or U-shaped deviations, confirming adequate fit of the BCPE distribution. As in the overall magnesium model, a modest upward drift is present in the positive quantile range of the younger age strata, consistent with mild residual positive skewness at lower fitted values. This pattern is expected given the biological constraints on pregnancy duration in younger, lower-risk women and does not materially affect the validity of the model or the interpretation of the marginal effect estimates. [alter_geburt: maternal age at delivery]

Online Resource 1e Analysis of association between overall magnesium supplementation and pregnancy duration: model quality - diagnostic plots

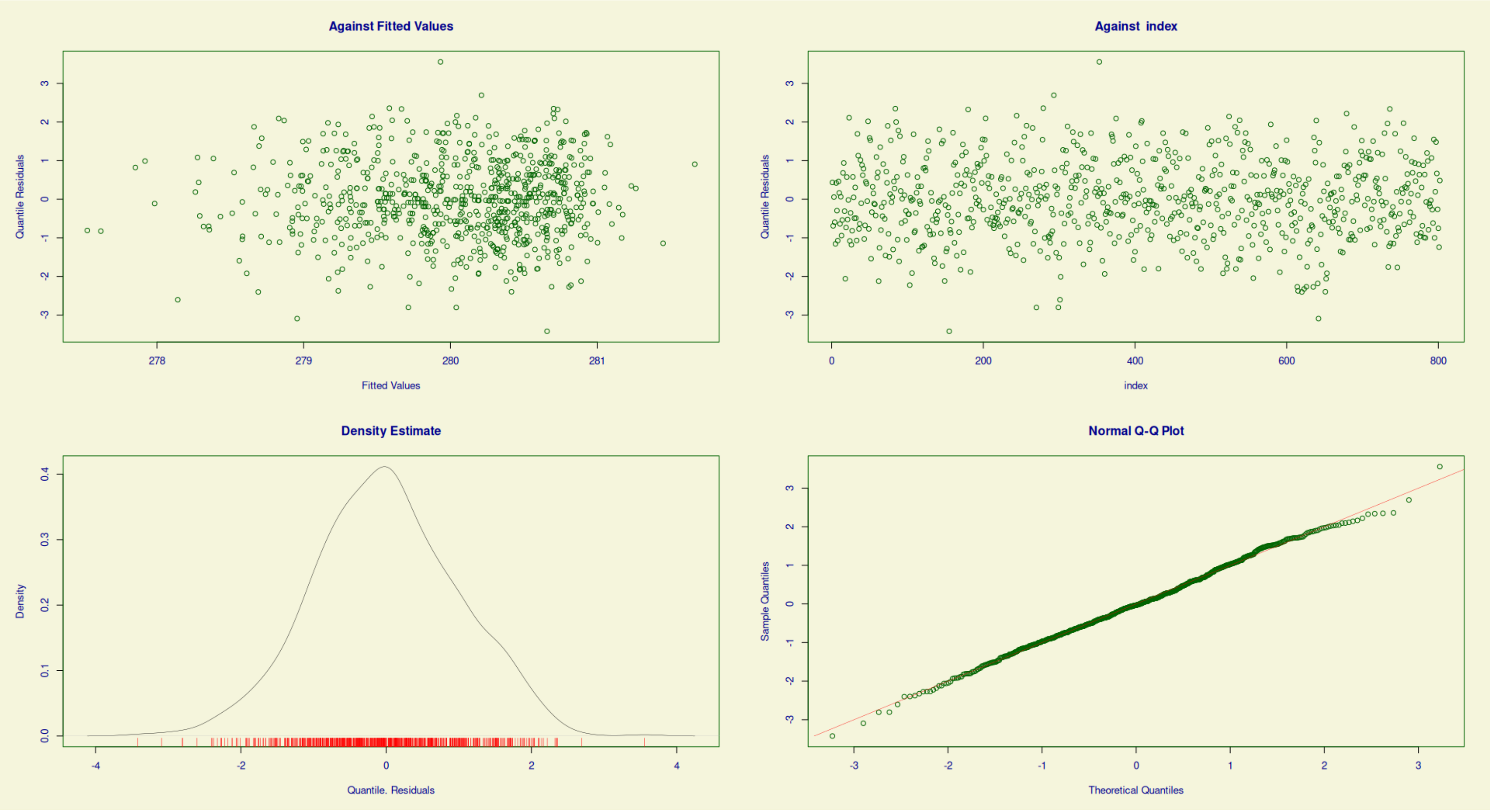

Randomised quantile residuals from the GAMLSS model for overall magnesium supplementation. Upper left: residuals plotted against fitted values (mu); no systematic pattern indicates adequate mean specification. Upper right: residuals plotted against observation index; absence of trend confirms no systematic variation related to data ordering or recruitment period. Lower left: kernel density estimate of residuals overlaid on the standard normal density; the close agreement indicates adequate distributional fit. Lower right: normal Q-Q plot of residuals; points follow the reference line closely across the central range, with minor deviations at the tails attributable to sparse observations at extreme pregnancy durations. Overall, the diagnostic plots support adequate model fit.

Online Resource 1f Analysis of association between mono-magnesium supplementation and pregnancy duration: model quality - diagnostic plots

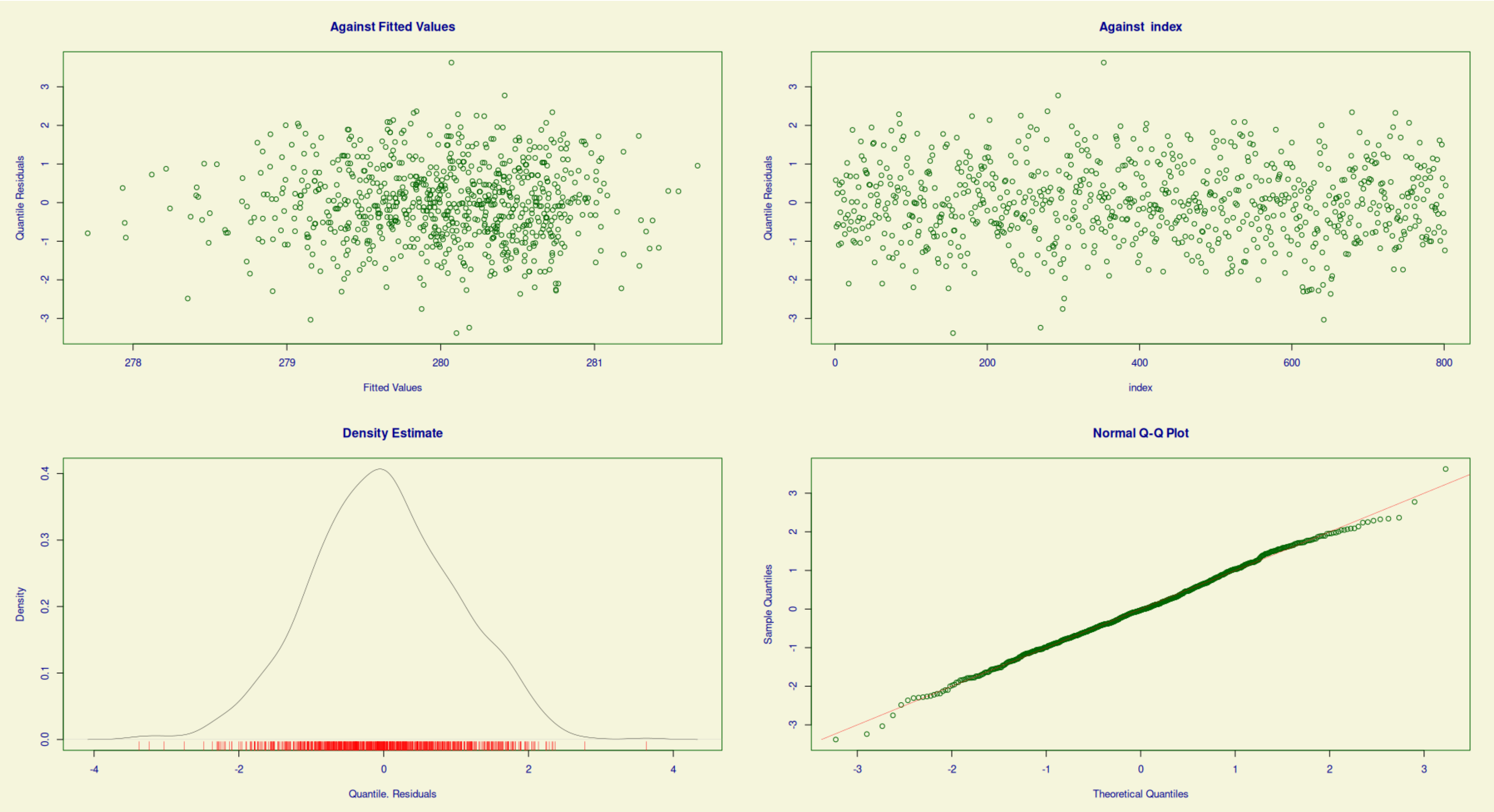

Randomised quantile residuals from the GAMLSS model for mono-magnesium supplementation. Panels as described in Online Resource X. The pattern of residuals is consistent with that observed for the overall magnesium model, with no systematic misspecification of the mean structure or distributional family. Minor tail deviations in the Q-Q plot are consistent with sparse data at extreme gestational ages and do not indicate model inadequacy.
