# Supplementary material for: High prevalence of magnesium supplementation during pregnancy: results from the LIFE Child study
Source: Arch Gynecol Obstet. 2026 Aug 31;313(1):261. doi: 10.1007/s00404-026-08522-z (PMC13529859; doi:10.1007/s00404-026-08522-z)
Supplement: Supplementary file 2 [file 404_2026_8522_MOESM2_ESM.pdf]

**High prevalence of magnesium supplementation during pregnancy: results from the LIFE Child study**

**Archives of Gynecology and Obstetrics**

Neininger*, Martina P[1,2]; Bremer*, Sophie UJ[1]; Dathan-Stumpf, Anne[3]; Stepan, Holger[3]; Kiess, Wieland[4,5]; Bertsche, Astrid[2,5]; Vogel, Mandy[4#]; Bertsche, Thilo[1#]

*) shared first authorship; #) shared senior authorship

Corresponding author:

Thilo Bertsche, Clinical Pharmacy, Institute of Pharmacy, Faculty of Medicine, Leipzig University and Drug Safety Centre, University Hospital Leipzig and Leipzig University, Bruederstrasse 32, 04103 Leipzig, Germany; email to thilo.bertsche@uni-leipizig.de, phone +49 341 97 11800; Fax number: +49 341 97 11849

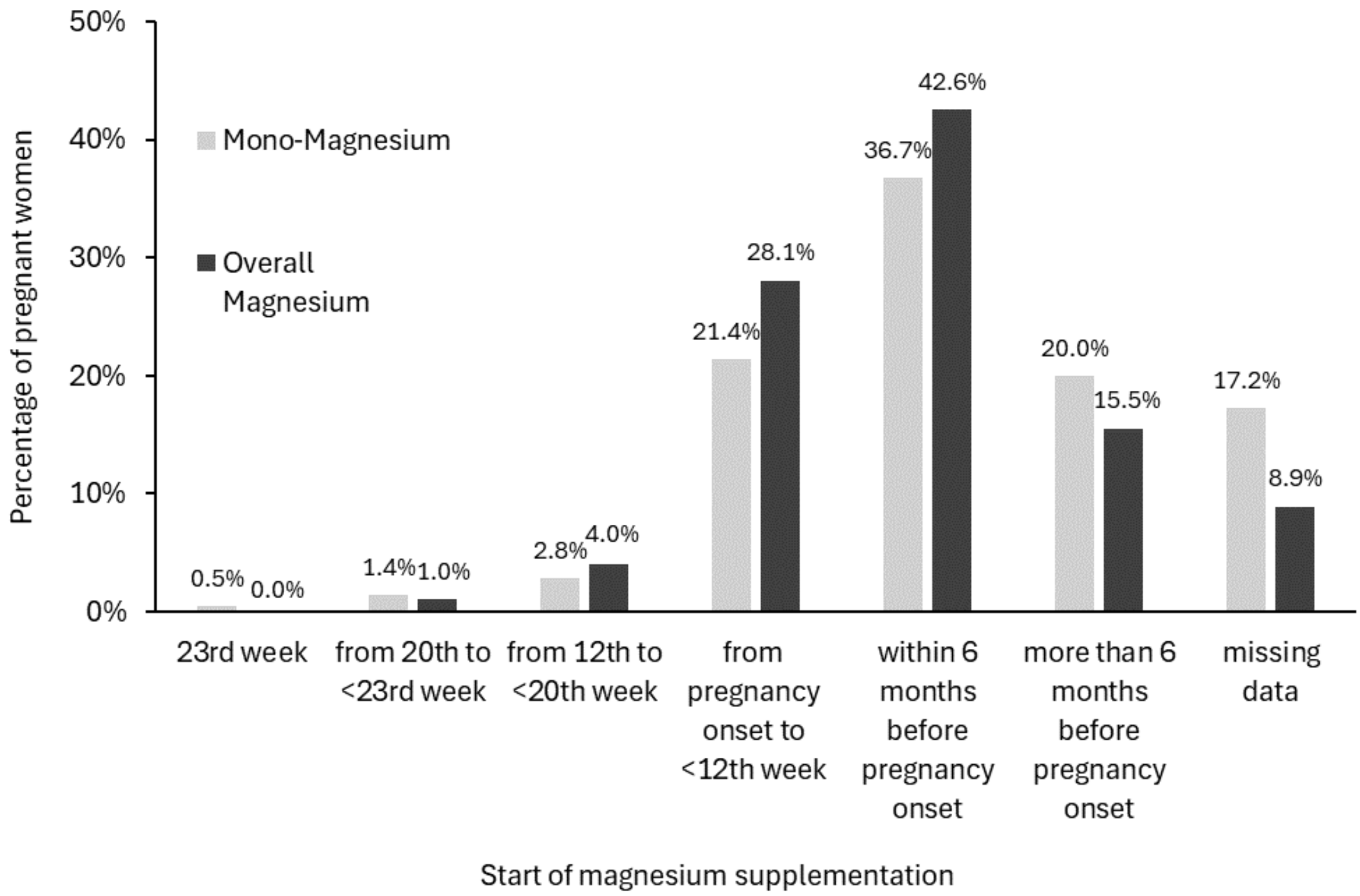


**Online Resource 2** Initiation of magnesium supplementation reported in the 24th gestational week
