# Supplementary material for: High prevalence of magnesium supplementation during pregnancy: results from the LIFE Child study
Source: Arch Gynecol Obstet. 2026 Aug 31;313(1):261. doi: 10.1007/s00404-026-08522-z (PMC13529859; doi:10.1007/s00404-026-08522-z)
Supplement: Supplementary file 3 [file 404_2026_8522_MOESM3_ESM.pdf]

High prevalence of magnesium supplementation during pregnancy: results from the LIFE Child study

Archives of Gynecology and Obstetrics

Neininger*, Martina P[1,2]; Bremer*, Sophie UJ[1]; Dathan-Stumpf, Anne[3]; Stepan, Holger[3]; Kiess, Wieland[4,5]; Bertsche, Astrid[2,5]; Vogel, Mandy[4#]; Bertsche, Thilo[1#]

*) shared first authorship; #) shared senior authorship

Corresponding author:

Thilo Bertsche, Clinical Pharmacy, Institute of Pharmacy, Faculty of Medicine, Leipzig University and Drug Safety Centre, University Hospital Leipzig and Leipzig University, Bruederstrasse 32, 04103 Leipzig, Germany; email to thilo.bertsche@uni-leipizig.de, phone +49 341 97 11800; Fax number: +49 341 97 11849

Online Resource 3 Characteristics of pregnancies with mono-magnesium supplementation with or without information on physician's prescription or recommendation

Characteristic	Magnesium-mono supplementation without information on prescription/ recommendation	Magnesium-mono supplementation with information on prescription/ recommendation	p-value
Total number [n]	432	215	-
Median gestational age at delivery (Q25; Q75) [weeks]	40+0 (39+0; 40+6) {n=365}	39+6 (38+6; 40+4) {n=154}	n.s.
Median age at birth (Q25; Q75) [years]	31.1 (28.4; 34.0) {n=409}	32.0 (29.2; 35.3) {n=181}	0.017
Socioeconomic status (SES) by Winkler-Stolzenberg index			
Median SES (Q25; Q75)	14.7 (12.3; 16.6) {n=413}	15.6 (12.6; 17.6) {n=201}	0.002*
SES Low	1.5% (6/413)	1.5% (3/201)	
SES Medium	60.3% (249/413)	51.7% (104/201)	n.s.*
SES High	38.3% (158/413)	46.8% (94/201)	
Geographic origin of the pregnant woman: Germany	95.1% (327/344)	93.1% (189/203)	n.s.
Median pre-pregnancy BMI (Q25; Q75) [kg/m^2]	22.5 (20.7; 24.8) {n=358}	22.9 (20.9; 25.3) {n=152}	n.s.
Obesity (BMI ≥30 kg/m^2)	6.9% (27/394)	6.5% (11/168)	n.s.
Primigravida	45.4% (186/410)	51.8% (87/168)	n.s.
Planned pregnancy	77.0% (281/365)	72.9% (151/207)	n.s.
(Gestational) Diabetes	6.0% (25/419)	10.2% (18/176)	n.s.
Premature contractions	7.9% (33/419)	3.4% (6/174)	n.s.
Hypertension	1.0% (4/419)	0.6% (1/174)	n.s.
Short inter-pregnancy interval (<12 months)	5.5% (20/361)	7.9% (12/152)	n.s.
Preterm birth in earlier pregnancy	3.2% (12/372)	5.0% (8/160)	n.s.
Infections	3.6% (15/419)	2.3% (4/174)	n.s.
Assisted pregnancy induction	4.6% (19/410)	7.8% (16/206)	n.s.
Preterm birth (< 37.0 weeks of gestation)	5.8% (21/365)	7.1% (11/154)	n.s.

BMI: body mass index, (Gestational) Diabetes: diagnosed using the IADPSG criteria for the oral glucose tolerance test, Hypertension: systolic blood pressure ≥140 mmHg, diastolic blood pressure ≥90 mmHg; The number of missing values varies between the variables, the numbers in curly brackets or in the denominator indicate the number of participants evaluated for the respective variable. A p-value ≤0.05 was considered to indicate significance.
*significant when used as continuous variable, not significant for comparison of grouped SES

The difference in age between the two sub-cohorts is in line with data from the federal government showing that the average age at birth increased from 30.6 years in 2011 to 31.5 years in 2019 [https://www.destatis.de/DE/Methoden/WISTA-Wirtschaft-und-Statistik/2012/12/geburten-sterbefaelle-ehe-2011_122012.pdf; https://www.destatis.de/DE/Presse/Pressemitteilungen/2020/07/PD20_282_122.html].
